# Preclinical Markers in Inflammatory Bowel Disease. A Nested Case–Control Study

**DOI:** 10.1093/crocol/otab072

**Published:** 2021-10-19

**Authors:** David Lundgren, Lovisa Widbom, Johan Hultdin, Pontus Karling

**Affiliations:** 1 Department of Public Health and Clinical Medicine, Division of Medicine, Umeå University, Umeå, Sweden; 2 Department of Medical Biosciences, Division of Clinical Chemistry, Umeå University, Umeå, Sweden

**Keywords:** albumin, biomarkers, calprotectin, C-reactive protein, Crohn’s disease, inflammatory bowel disease, ulcerative colitis

## Abstract

**Background:**

Our objective was to determine if patients who later develop inflammatory bowel disease (IBD) show signs of increased inflammatory activity in plasma measured with high sensitivity C-reactive protein (CRP), calprotectin, and albumin before the clinical onset of IBD.

**Methods:**

We identified 96 subjects who later developed IBD (70 ulcerative colitis [UC] and 26 Crohn’s disease [CD]). High sensitivity CRP, calprotectin, and albumin were analyzed in frozen plasma, donated from cases and sex–age matched controls 1–15 years before diagnosis.

**Results:**

We found that subjects who later developed UC had lower albumin levels, and subjects who later developed CD had higher CRP levels than controls. Multivariable conditional logistic regression with albumin, calprotectin, and CRP showed a lower risk for developing IBD and UC with higher albumin levels (odds ratio [OR] 0.79, confidence interval [CI] 0.69–0.90; respective OR 0.77, CI 0.66–0.91). Higher CRP levels were associated with an increased risk of developing CD (OR 1.314, CI 1.060–1.630). When adjusting for body mass index or smoking in the logistic regression model, similar results were found. Plasma calprotectin levels in the preclinical period among patients with IBD did not differ from controls.

**Conclusions:**

In this nested case–control study, subjects who later developed IBD had signs of low-grade systemic inflammation, indicated by significantly higher CRP plasma levels in CD and lower albumin plasma levels in UC, before the onset of clinical disease.

## Graphical Abstract

**Figure:**
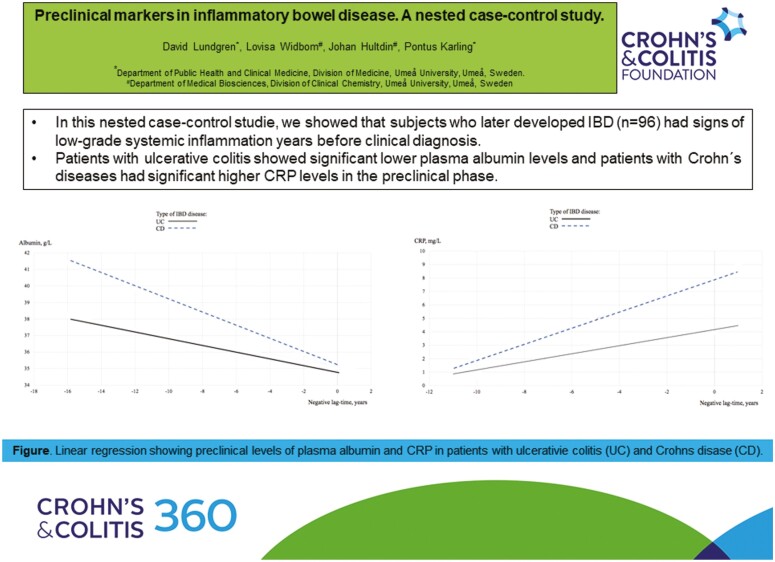

## Introduction

Inflammatory bowel disease (IBD) is a chronic relapsing inflammatory disorder with a prevalence exceeding 0.3% in the Western population.^1^ IBD commonly presents early in life, but approximately 1 in 3 patients with ulcerative colitis (UC) and 1 in 4 patients with Crohn’s disease (CD) develop the disease after the age of 40, referred to as “late-onset disease.”^2^ The cause of IBD is unknown, but the pathogenesis involves a dysfunctional interaction between epithelial cells, immune cells, and microbiota, influenced by genetic and environmental factors.^3,4^ In most cases, the onset of IBD is insidious, and like other immune-mediated diseases, there is evidence that the onset of symptoms in IBD is preceded by a preclinical phase.^5^ The incidence of IBD is increasing in developing countries and an increasing number of patients with IBD will be a challenge for the healthcare systems in the future. Therefore, to optimize the early interventions for patients with IBD it is important to achieve more knowledge on the patient disease behavior in the preclinical phase.^6^

Studies have shown evidence of IBD-related serological markers in the preclinical phase in patients with IBD as well as biomarkers of inflammation.^7–9^

Commonly used biomarkers in clinical practice to diagnose and monitor disease activity in IBD are C-reactive protein (CRP), serum or plasma albumin, and fecal calprotectin.^10^ The production of CRP in the liver is triggered by cytokines (ie, interleukin-6, tumor necrosis factor, and interleukin-1β) from gut inflammation^11^ and albumin is depressed in the acute-phase response.^12^ Calprotectin is a protein found in high concentrations in neutrophil granulocytes. It increases in plasma, urine, cerebral spinal fluid, and stool in processes that cause increased neutrophil activity.^13,14^ It has antibacterial properties, induces apoptosis, promotes inflammation, and may increase more than 100-fold in inflammatory conditions.

Fecal calprotectin shows high sensitivity and a high negative predictive value for intestinal inflammation and is used to discriminate irritable bowel syndrome from IBD and for disease monitoring in IBD.^15,16^ Calprotectin in serum or plasma also rises in parallel with inflammatory activity in patients with IBD, and previous studies show that calprotectin in serum or plasma performs equally or better to predict inflammation compared to CRP and albumin.^17–20^

There is uncertainty about when and how the inflammatory process is triggered in IBD and at what stage in the preclinical period systemic inflammation can be detected.

This study aims to determine if patients who later develop IBD show signs of systemic inflammation measured by high-sensitive CRP, calprotectin, and albumin years before the clinical onset of IBD.

## Materials and Methods

### Study Population

All subjects included in this nested case–control study were recruited from 2 large health surveys; the “Västerbotten Intervention Programme (VIP)” and the “Mammography screening project (MA).” VIP has since 1985 annually invited subjects in Västerbotten County, Sweden at 40, 50, and 60 years of age (6500–7000 subjects every year).^21^ VIP includes blood samples and validated questionnaires that focus on lifestyle issues. The MA project includes women aged 18–82 years who donated blood samples simultaneously with mammary screening between 1995 and 2006. In total, approximately 54000 blood samples have been donated in the MA project.

Case identification was performed by searching for ICD codes (K50.1-9 for CD and K51.1-9 for UC) within VIP and MA. All the identified patients with an ICD code for IBD were then thoroughly evaluated in a medical chart review from journals of the departments of medicine, surgery, radiology, and pathology to confirm correct IBD diagnosis according to the Montreal classification.^22^ The date of diagnosis was defined as the date of the first endoscopic investigation or diagnostic imaging showing signs of IBD.

In the initial search, 121 patients were identified. Patients diagnosed with IBD within 1 year after blood samples were collected were excluded. After exclusion, 70 patients with UC and 26 patients with CD were included in the study. For each patient with IBD, 2 controls matched for age, gender, health survey, area of residence, and inclusion date were randomly identified in VIP and MA.

### Sample Collection and Handling

Blood samples were collected in Na-heparin tubes (10mL), inverted 8–10 times directly following sampling. The tubes were centrifuged at 1500 G for 15 minutes, and plasma was frozen in aliquots within 1 hour and stored at −80 °C.

### Biochemical Analyses

All plasma samples were analyzed in batch in triplets with 1 case and its matched controls in random order blinded to the technicians. All samples were analyzed at a single center, the accredited Department of Laboratory Medicine, Clinical Chemistry, University Hospital Umeå.

Albumin and CRP were analyzed in plasma on a Cobas 8000 modular analyzer (Roche Diagnostics GmbH). Reagents used were Albumin BCP 400 (Cat. No. 05975573190) and CRPL3 (Cat. No. 05172373190) from Roche Diagnostics. Albumin was standardized to reference preparation ERM DA470k. CRP is traceable to CRM 470 (CRPL3 2011-01, V3).

The total coefficients of variation were for albumin 5% and 3% at levels of 25 and 60g/L, respectively, and for CRP 1.5% and 1.9% at levels of 8 and 47mg/L, respectively.

Calprotectin in plasma was analyzed with the quantitative CalproLab ELISA TEST (ALP), product No. CALP0170 (CALPRO AS). The lowest level of detection was 5 µg/L. The within series coefficients of variations were 5.31%, 6.11%, and 8.75% at levels 287, 1138, and 3802 µg/L, respectively.

### Statistical Analyses

IBM SPSS version 24.0 was used for statistical analyses. Basal characteristics were presented as median (25th–75th percentile). Mann–Whitney *U*-test was used for continuous variables, Chi^2^ test was used for categorical variables, and Spearman’s rho was used for correlations. A *P* value <0.05 was considered statistically significant.

**Figure 1.**
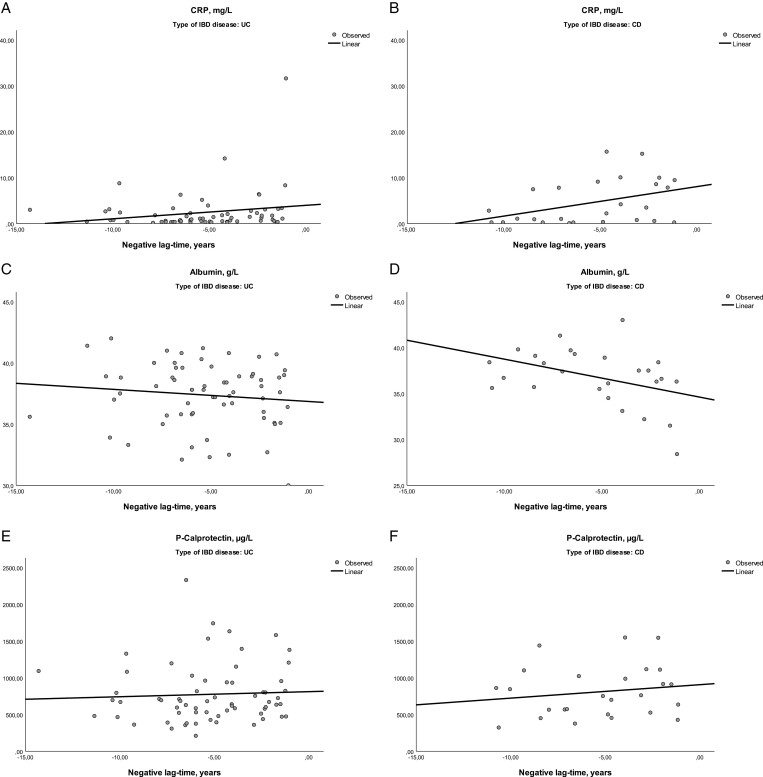
(A–F) Levels of CRP, albumin, and calprotectin levels in the preclinical phase of ulcerative colitis (UC) and Crohn’s disease (CD). Abbreviation: CRP, C-reactive protein.

Univariate and multivariate conditional logistic regression was performed for CRP, albumin, calprotectin, body mass index (BMI), and smoking. Linear regressions and related plots were used to assess correlations for CRP, albumin, and calprotectin with lag time to diagnosis (Figure 1).

### Ethical Considerations

The project was approved by the committee for human ethics at Umeå University (Dnr 06-024M, 2010-284-31M).

## Results

Table 1 shows basal characteristics for cases and controls. Smoking was more common among subjects who later developed IBD, compared to controls (31% vs. 14%; *P* = 0.041). Albumin was significantly lower in subjects who later developed UC, and high-sensitive CRP was significantly higher in subjects who later developed CD, whereas there were no differences in calprotectin between cases and controls.

**Table 1.** Baseline characteristics for ulcerative colitis and Mb Crohn with matched controls.

Ulcerative colitis	Case	Control	*P* ^a^	*N* case/control
Age, years	50.1 (40.0–59.8)	50.1 (40.1–59.7)	.86	70/139
Lag time to diagnosis, years	5.26 (2.66–7.23)	n.a.	n.a.	70/n.a.
Gender, women	61.4	54.7	.77	70/139
BMI, kg/m^2^	25.0 (23.2–27.5)	25.6 (23.1–27.8)	.82	70/138
Smoking	30.0	20.1	.16	65/128
Albumin, g/L	37.8 (35.8–39.1)	38.5 (36.6–39.8)	.025†	65/139
Calprotectin, µg/L	671.4 (496.4–946.7)	693.2 (494.6–909.4)	.93	65/137
CRP, mg/L	1.08 (0.46–2.70)	0.94 (0.49–2.52)	.69	65/139
Mb Crohn	Case	Control	*P* ^a^	*N* case/control
Age, years	50.2 (40.1–56.8)	50.0 (40.2–59.7)	.86	26/52
Lag time to diagnosis, years	4.76 (2.50–8.08)	n.a.	n.a.	26/n.a.
Gender, women	46.2	50.0	.94	26/52
BMI, kg/m^2^	26.1 (23.1–30.4)	25.3 (22.9–28.3)	.43	26/52
Smoking	34.6	17.3	.18	22/42
Albumin, g/L	37.1 (35.6–39.0)	38.0 (36.2–40.2)	.07	26/52
Calprotectin, µg/L	756.9 (520.4–1042.5)	639.8 (464.2–924.8)	.37	26/52
CRP, mg/L	2.51 (0.34–8.71)	0.83 (0.31–2.10)	.018^†^	26/52

Median (25%–75%) for continuous variables, proportions (%) for noncontinuous variables. Abbreviations: BMI, body mass index; CRP, C-reactive protein; n.a., not applicable.

^a^Calculated with Mann–Whitney *U*-test for continuous variables and Chi^2^ for categorical variables.

*P* < .05.

There was a significant Spearman correlation between high-sensitive CRP and albumin, calprotectin, and BMI (−0.299, 0.389, and 0.283, respectively, *P* value <.001 for all). Albumin showed a significant correlation with smoking (0.207; *P* = .001).

### Conditional Logistic Regression

Univariable conditional logistic regression showed a significantly higher risk of developing UC and CD with lower albumin (Table 2). Also, elevated CRP levels were significantly associated with a higher risk for developing CD. There was no association between calprotectin levels and IBD. Using log-transformed variables did not change the result.

**Table 2.** Univariable conditional logistic regression, odds ratio (OR), and 95% confidence interval (CI).

	IBD	UC	CD	*N* case/control
Albumin	0.797 (0.707–0.899)∗	0.805 (0.696–0.932)∗	0.780 (0.631–0.965)∗	91/181, 65/129, 26/52
Calprotectin	1.000 (0.999–1.000)	1.000 (0.999–1.000)	1.000 (0.999–1.001)	91/179, 65/127, 26/52
CRP	1.052 (0.988–1.121)	1.007 (0.948–1.070)	1.329 (1.094–1.614)∗	91/181, 65/129, 26/52

Abbreviations: CD, Crohn’s disease; CRP, C-reactive protein; IBD, inflammatory bowel disease; UC, ulcerative colitis.

∗Statistically significant.

In the multivariate analysis with the variables CRP, albumin, and calprotectin, normal albumin was protective for overall IBD and UC, and the protective effect remained after adding BMI and smoking in the model (Table 3). An elevated CRP was significantly associated with a higher risk for CD but not UC. Calprotectin levels did not differ between subjects who later developed IBD and controls, but adding BMI and smoking in the model showed that increased calprotectin levels were protective for overall IBD.

**Table 3.** Multivariable conditional logistic regression, odds ratio (OR), and 95% confidence intervals (CIs).

	IBD	UC	CD	*N* case/control
Model 1				91/179, 65/127, 26/52
Albumin	0.789 (0.691–0.901)∗	0.773 (0.657–0.909)∗	0.877 (0.667–1.153)	
Calprotectin	1.000 (0.999–1.000)	1.000 (0.999–1.000)	0.999 (0.998–1.001)	
CRP	1.020 (0.955–1.089)	0.982 (0.920–1.048)	1.314 (1.060–1.630)∗	
Model 2				91/178, 65/126, 26/52
Albumin	0.788 (0.690–0.899)∗	0.769 (0.654–0.904)∗	0.876 (0.665–1.154)	
Calprotectin	1.000 (0.999–1.000)	1.000 (0.999–1.000)	0.999 (0.998–1.001)	
CRP	1.014 (0.947–1.086)	0.970 (0.900–1.045)	1.323 (1.046–1.675)∗	
Model 3				82/157, 60/115, 22/42
Albumin	0.793 (0.685–0.918)∗	0.747 (0.621–0.898)∗	0.925 (0.687–1.247)	
Calprotectin	0.999 (0.999–<1.000)∗	0.999 (0.999–1.000)	0.999 (0.998–1.001)	
CRP	1.021 (0.952–1.095)	0.973 (0.899–1.053)	1.281 (1.030–1.593)∗	

Model 1: albumin, calprotectin, and CRP. Model 2: Model 1 + BMI. Model 3: Model 2 + smoking. Abbreviations: CD, Crohn’s disease; CRP, C-reactive protein; IBD, inflammatory bowel disease; UC, ulcerative colitis.

∗Statistically significant.

### Linear Regression

Linear regression models assessing the relation between the time before diagnosis and levels of inflammatory biomarkers showed a statistically significant decline in albumin levels (*r* square 0.164; *P* = .040) and a rise of CRP levels (*r* square 0.163; *P* = .041) in the preclinical phase for the patients that developed CD. There was no statistically significant rise in calprotectin in patients with IBD in the preclinical phase (Figure 1A–F).

## Discussion

There is uncertainty about what triggers IBD and how the disease evolves in the preclinical phase, but it has been assumed that there is a period of low-grade inflammation before the onset of symptoms in IBD.^5^ This study focuses on common biomarkers of inflammation that are used in clinical practice. A rise of some of these markers proceeds flares in established disease. For example, a rise in fecal calprotectin can be detected sometimes months before a flare.^23^ Therefore, it is reasonable to assume that biomarkers of inflammation would be detected in the preclinical phase.

In this nested case–control study, we found that patients who later developed UC and CD had lower albumin levels in the preclinical phase. Also, a rise in CRP levels was found in patients later developing CD. However, for plasma calprotectin, no difference was seen.

In another nested case–control study, Lochhead et al demonstrated that high levels of high-sensitive CRP and interleukin-6 were associated with a risk of incident CD and UC in a cohort of predominately female subjects.^9^ However, higher CRP levels in our study were only seen in patients later developing CD. In general, CD patients show higher CRP levels than patients with UC at diagnosis.^24,25^ Contrary to the CRP rise toward a diagnosis in our study, Lochhead et al could not detect a correlation between CRP and time to diagnosis.

Fecal calprotectin is a sensitive marker for IBD,^15,16^ but also, an increase in serum calprotectin has been shown in active IBD. In rheumatoid arthritis, serum calprotectin is an excellent and sensitive marker for disease activity, correlates to disease severity, and predicts flares of disease.^26–28^ Therefore, we aimed to test if an increase in plasma calprotectin could be shown before the clinical onset of IBD. However, our study shows that both in patients that later developed UC or CD, plasma levels of calprotectin were equal to control subjects in the preclinical phase.

In comparison to CRP and albumin, calprotectin seems not to respond to preclinical disease. CRP is a true acute-phase protein produced in hepatocytes in response to tumor necrosis factor, interleukin-1, and interleukin-6,^26^ whereas calprotectin is mainly an intracellular protein in the cytosol of neutrophils, and elevated calprotectin levels mirrors increased neutrophil activity.^13,14^ Perhaps, the neutrophil activity, especially in blood, or the transmission of calprotectin from the gut to blood, or both, is a later event in the evolution of IBD. However, a normal calprotectin does not exclude that our patients that later developed IBD had an elevated fecal calprotectin in the preclinical phase. For example, there is evidence that asymptomatic twins to IBD patients have increased levels of fecal calprotectin.^29–31^

The protective effect of high calprotectin levels on IBD when adding BMI and smoking into the logistic regression model in our study was an unexpected finding. The association needs to be replicated in future studies.

The lower levels of albumin in patients that later developed IBD in our study may represent a low-grade chronic inflammatory state in these subjects. Hypoalbuminemia is considered mainly to be a consequence of the extent of physiological stress rather than undernutrition in patients with chronic disease.^32^ Hypoalbuminemia in chronic disease is believed to be caused by a combination of an inflammatory response and an increased capillary permeability. In addition, inflammation increases albumin breakdown in the liver.^33^ A low level of albumin is an indicator of more severe inflammatory activity, and hypoalbuminemia at UC diagnosis was a predictor of more severe disease in 1 study.^34^ In contrast to CRP, which drops quickly after an inflammatory burst, albumin normalizes much slower (sometimes months) after an acute inflammatory response.^32^ The prolonged effect of inflammation on albumin might make hypoalbuminemia a better marker of low-grade chronic inflammation than, for example, an elevated CRP or calprotectin.

Other serological markers than those presented in our study have been analyzed in the preclinical phase in previous studies. Circulating antibodies are detected in the subclinical phase of other chronic immune-mediated diseases and have been associated with an upregulation of several cytokines and other inflammatory proteins.^5,35^ The serological markers; antineutrophil cytoplasmic antibodies (pANCA), and the antimicrobial antibodies; anti-*Saccharomyces cerevisiae* (ASCA), anti-Cbir1, anti OmpC, anti-A4-Fla2, and anti-FlaX have been detected in patients with IBD years before diagnosis.^7,8,36,37^ The presence of these serological markers in blood has been associated with disruption of the intestinal mucosa barrier and therefore represents a possible immunological response to microbes in the gut.^38^ Interestingly, some of these serological markers have been linked to a more aggressive phenotype in CD.^36^

There are some limitations to our study. In this study, we rely on only 1 sample for cases and controls. Ideally, a prospective study sampling subjects at different time points would have been preferable. For other chronic immune-mediated diseases, there are such large-scale longitudinal studies ongoing.^39–41^ However, longitudinal “screening studies” for IBD are challenging to perform due to the large sample and the long observation time needed. Also, the number of patients in this study is small (especially patients with CD), making the study sensitive for Type I errors. The CRP concentrations in cases and controls are in the low range of the method. This could lead to a higher imprecision and possibly blur differences between cases and controls. Still, we found significant differences. The 2 large health surveys that were the base of our study were mainly constructed to explore risk factors for cardiovascular disease and cancer, therefore only blood samples and not fecal samples were collected. Fecal calprotectin is known to be a better biomarker to discriminate IBD from controls^19^ and probably a better marker to use when study preclinical calprotectin levels.

Finally, the patients with IBD in our study are older at onset than average IBD patients, and therefore these results might not be generalizable to younger patients.

In conclusion, in this nested case–control study, subjects who later developed IBD show signs of low-grade systemic inflammation in the preclinical phase. In patients with CD the plasma levels of CRP increased and in patients with UC the plasma levels of albumin decreased the years before diagnosis. There were no changes in plasma calprotectin levels before diagnosis.

## Data Availability

Data not publically available but can be requested from the corresponding author.
